# Straw retention efficiently improves fungal communities and functions in the fallow ecosystem

**DOI:** 10.1186/s12866-021-02115-3

**Published:** 2021-02-17

**Authors:** Caifang Zhang, Zhaoli Lin, Youxiong Que, Nyumah Fallah, Muhammad Tayyab, Shiyan Li, Jun Luo, Zichu Zhang, Ahmad Yusuf Abubakar, Hua Zhang

**Affiliations:** 1 Key Laboratory of Sugarcane Biology and Genetic Breeding, Ministry of Agriculture, Fujian Agriculture and Forestry University, Fuzhou, 350002 China; 2 College of Agriculture, Fujian Agriculture and Forestry University, Fuzhou, 350002 China; 3 Fuzhou No.8 High School, Fuzhou, 350000 China

**Keywords:** Sugarcane straw retention, Soil profile, Fungal community, Network analysis, FUNGuild analysis

## Abstract

**Background:**

Straw retention is a substitute for chemical fertilizers, which effectively maintain organic matter and improve microbial communities on agricultural land. The purpose of this study was to provide sufficient information on soil fungal community networks and their functions in response to straw retention. Hence, we used quantitative real-time PCR (qRT-PCR), Illumina MiSeq (ITS rRNA) and FUNGuild to examine *ITS rRNA* gene populations, soil fungal succession and their functions under control (CK) and sugarcane straw retention (SR) treatments at different soil layers (0–10, 10–20, 20–30, and 30–40 cm) in fallow fields.

**Result:**

The result showed that SR significantly enhanced *ITS rRNA* gene copy number and Shannon index at 0–10 cm soil depth. Fungi abundance, OTUs number and ACE index decreased with the increasing soil depth. The ANOSIM analysis revealed that the fungal community of SR significantly differed from that of CK. Similarly, significant difference was also observed between topsoil (0–20 cm) and subsoil (20–40 cm). Compared with CK, SR decreased the relative abundance of the pathogen, while increased the proportion of saprotroph. Regarding soil depth, pathogen relative abundance in topsoil was lower than that in subsoil. Besides, both sugarcane straw retention and soil depths (topsoil and subsoil) significantly altered the co-occurrence patterns and fungal keystone taxa closely related to straw decomposition. Furthermore, both SR and topsoil had higher average clustering coefficients (aveCC), negative edges and varied modularity.

**Conclusions:**

Overall, straw retention improved α-diversity, network structure and fungal community, while reduced soil pathogenic microbes across the entire soil profile. Thus, retaining straw to improve fungal composition, community stability and their functions, in addition to reducing soil-borne pathogens, can be an essential agronomic practice in developing a sustainable agricultural system.

**Supplementary Information:**

The online version contains supplementary material available at 10.1186/s12866-021-02115-3.

## Background

Fertilization is a crucial agricultural approach that not only improves plant nutrient storage but also simultaneously alters soil attributes and microbial communities [1–3]. In the past few decades, extensive fertilization, especially nitrogen fertilizer, has been used to raise sugarcane production to meet the growing sugar demand [4]. Although inorganic fertilization has a positive effect on sugarcane yield, on the other hand, it has unfavorable indirect effects on soil quality by causing soil acidification, enhancing soil pathogens, intensifying nitrification and leaching of nitrates [4–6]. In contrast, organic fertilization is an alternative approach to chemical fertilization to mitigate soil acidification and to improve soil nutrient status, thus ensuring sugarcane productivity [7–9].

Straw retention (SR) has an important role in soil aggregation, and nutrient availability, increasing soil microbial biomass and functional diversity [10, 11]. Therefore SR is a preferred approach for better agricultural, environmental sustainability, and global biogeochemical cycles [12–14]. The soil microbiome plays a pivotal role in soil ecosystem process and is an important driving force for the biogeochemical cycle of basic elements such as nitrogen (N) and carbon (C). In particular, fungi play an essential role in the successful biotransformation of organic substrates in straw retention ecosystem. Topsoil (0–20 cm) holds greater microbial biomass and diversity. In contrast, a high subsoil volume (below 20 cm) on a depth-weighted basis also causes much microbial abundance and diversity [15–19]. Soil microbial composition changes with increasing soil depth, while microbial diversity generally declines with depth [20, 21]. Saprophytic fungi greatly participate in the nutrient cycle in terrestrial ecosystems, while symbiotic fungi are beneficial to the health, nutrition and quality of most crops [22]. Research has shown that rice straw input positively impacts soil biogeochemistry and can improve soil fertility and fungal community diversity [23].

High throughput sequencing (HTS) has been employed to investigate fungal community composition in soil, however, our understanding of the function and network of fungal community in a fallow straw retention system is very limited [24]. Furthermore, no-tillage and traditional agricultural systems have significant differences in soil fungal communities [25], but little is known about fungal communities’ response to soil depth in sugarcane cultivation system.

FUNGuild is a novel tool for estimating functional diversity of fungal communities and also for comprehensively exploring fungal communities from an ecological perspective [26]. The fungal OTUs from HTS can be apportioned into 3 trophic modes and 12 guilds based on a database. LaMondia et al. documented that straw mulch did not affect the potato early dying disease, nematodes, or tuber yield [27]. However, many researchers reported that straw retention can enhance the soil’s ability to resist major plant diseases [28, 29]. For example, Donovan et al. mentioned that crop residues retention increased soil ability to resist the presence of crown rot of wheat [28]. Therefore, the importance of fungal pathogens to animals and plants can not be ignored. Many researchers have extensively studied the effect of straw retention on the composition of soil microbial communities in agricultural soils [30–32]. However, understanding of the unusual fungal diversity and its complex interactions with crop residues in farmland is still limited. The interaction of fungi with soil carbon and sugarcane roots in different soil profiles favors fungal taxa with diverse life-history strategies. For instance, the abundance of plant roots and carbon in soil surface can produce a diverse symbiotrophs and saprotrophs communities, while subsoil, which are relatively low in carbon, can select discrete symbiotrophs and pathotrophs communities. In addition, shifts in fungal community composition and diversity in different soil profiles can be reflected in the unique species patterns and interactions in the fungal network.

Network analysis is essential to understand better the complex webs of fungus associations, which provides crucial insights into biological systems. Information on network structure (topology) is used to categorize “hub” species that are associated with many other species within networks depicting multiple species host-symbiont relationships [33–35]. These hubs with broad host/symbiont ranges are essential for modulating different ecological processes within a community [36, 37]. Researchers have used HTS technology to record information about fungal communities associated with plants [38, 39]. Identifying the hub microbial species among thousands of other species in the network has become a significant approach to understand ecosystem-scale phenomena.

Therefore, more understanding of fungal distribution patterns in soil profiles and fundamental mechanisms must update our current knowledge and future predictions of straw retention function. Accordingly, we hypothesize that soil depth and straw retention play a crucial role in changing the fungal community composition, network structure and trophic modes of keystone taxa. To investigate this, we analyzed soil fungal communities in response to straw retention in different soil profiles using MiSeq sequencing of fragments of the fungal internal transcriptional spacer (ITS).

## Results

### qRT-PCR

**Fig. 1:**
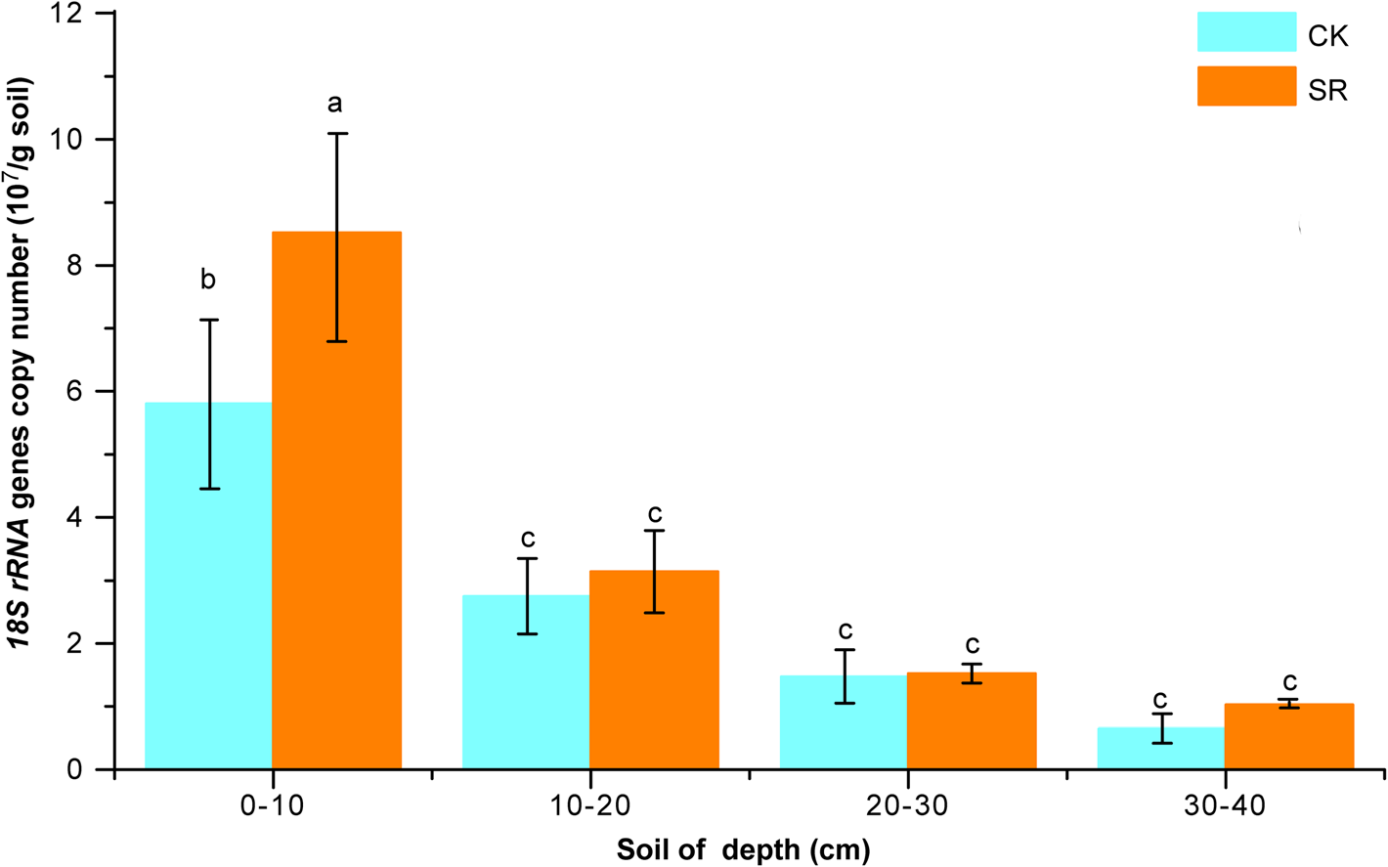
The copies number of *ITS rRNA* gene in four soil depths (0–40-cm) under sugarcane straw retention (SR) treatment compared to control (CK). Error bars in the histogram with different lowercase letters show significant differences between treatments (Tukey test, *n* = 3, *p* < 0.05)

The qRT-PCR results of fungal *ITS rRNA* gene copies showed that exponentially distributed fungal communities on both CK and SR along with the soil depth. In soil depth, 0–10 cm, the number of *ITS rRNA* copies in SR was significantly higher than CK (*p* < 0.05). Meanwhile, both CK and SR treatments in 0–10 cm soil layer were significantly higher than in other soil layers (10–20, 20–30 and 30–40 cm) (*p* < 0.05) (Fig. 1). Two-way ANOVA interaction analysis showed that soil depth was the main factor affecting fungal abundance (Table S2).

### Alpha diversity

A total of 960,252 (average of 40,011) filtered fungal readings were obtained from each soil sample (Table S3). Additionally, Good’s coverage values ranged from 98 to 99% at 97% similarity cutoff. The result indicated that there were sufficient sequence reads to capture fungal richness and diversity from all soil samples. Compare to CK, OTUs number and ACE index of SR did not show a significant difference in all layers, however, the Shannon index was higher in 0–10 cm soil depth (*p* < 0.05) (Table 1). In CK and SR, the OTUs number and ACE index reduced with soil depth. However, in SR treatment, the OTUs number was higher in 0–10 cm than 30–40 cm soil depth (*p* < 0.05), while the ACE index in soil layer 0–10 cm was significantly higher relative to that in 10–20 cm and 30–40 cm soil depths (*p* < 0.05). Multivariate ANOVA analysis revealed that soil depths significantly influence OTUs number and ACE index (*p* < 0.05) (Table 1).

**Table 1:** Alpha diversity index

Treatment	Depth	OTUs_num	ACE_index	Shannon_index	Coverage
CK	0–10	1519.33 ± 87.82ab	2153.91 ± 70.83ab	3.96 ± 0.34c	0.99
CK	10–20	1612.33 ± 68.60ab	2130.15 ± 70.47ab	4.68 ± 0.23abc	0.99
CK	20–30	1541.00 ± 91.15ab	1932.04 ± 167.98abc	4.69 ± 0.60abc	0.99
CK	30–40	1041.33 ± 140.88c	1511.80 ± 126.94c	4.83 ± 0.15abc	0.99
SR	0–10	1671.33 ± 22.70a	2202.85 ± 50.09a	5.11 ± 0.15a	0.98
SR	10–20	1332.33 ± 102.49abc	1635.66 ± 136.23bc	5.02 ± 0.12ab	0.99
SR	20–30	1343.67 ± 100.75abc	2008.35 ± 184.09abc	4.88 ± 0.27abc	0.98
SR	30–40	1261.00 ± 273.58bc	1548.86 ± 386.96c	4.12 ± 0.38bc	0.99
	Treatment	0.08	0.43	1.12	
	Depth	4.15^a^	4.51^a^	0.68	
	Treatment×Depth	1.82	1.17	2.93	

Alpha diversity index at 0–40-cm depth under sugarcane straw retention (SR) treatment compared to control (CK). Different letters indicate significant differences between samples (Tukey test, *p* < 0.05). Values are mean ± standard errors (*n* = 3). Treatment: CK SR. Depth: soil of depth in 0–10, 10–20, 20–30, 30–40 cm. Multivariate ANOVA for the effects of straw retention and soil depth on number of OTUs number, ACE index, Shannon index

^a^represent the level of significance at 0.05

### Relative abundance of dominant Phyla

**Fig. 2:**
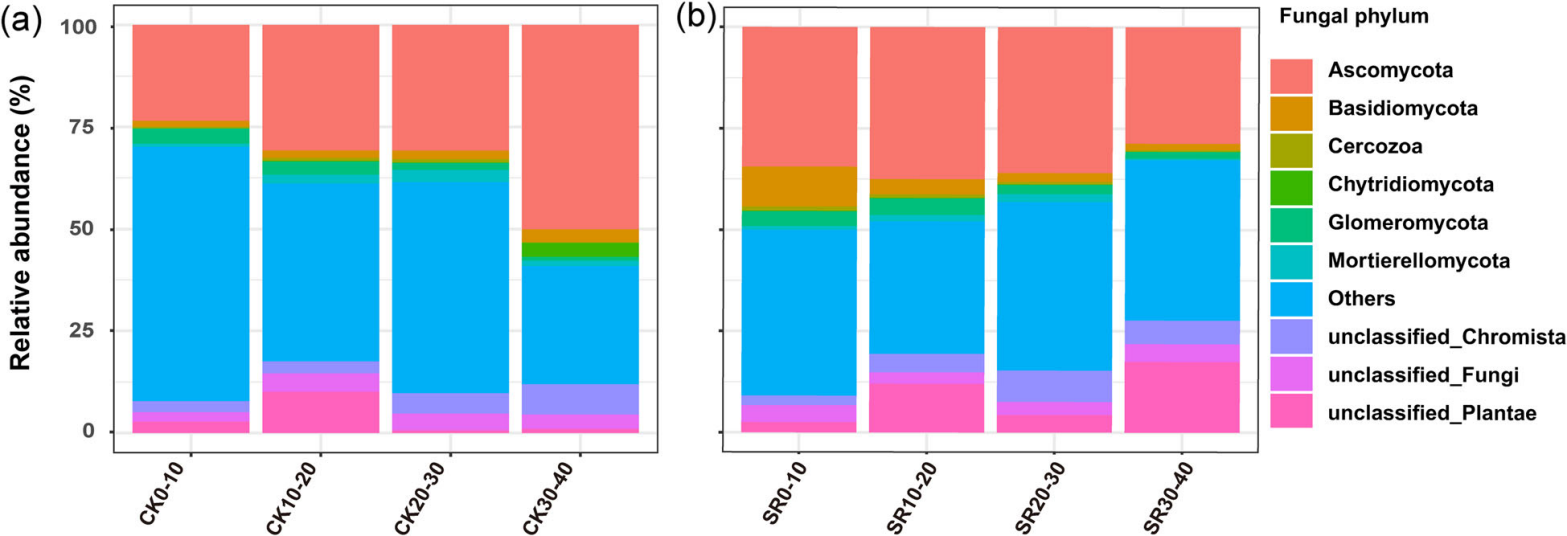
Relative abundances of the top 10 fungal phyla (relative abundance > 0.04%) at different depths, **a** in control (CK) treatments, and **b** sugarcane straw retention (SR). “Others” refers to those identified phyla that were beyond the top 10 phyla. CK, control; and SR, sugarcane straw retention

The relative abundance of Ascomycota (23.4–50.0%) and Basidiomycota (1.4–9.8%) was higher in the soil layer (0–40 cm), followed by Glomeromycota (1.0–4.0%), Mortierellomycota (0.4–3.0%) and Chytridiomycota (0.0–3.4%) (Fig. 2). In CK treatment, the phylum Ascomycota was enhanced with increasing soil depths (Fig. 2a). However, the abundance of Ascomycota in SR treatment was evenly distributed in various soil depths, ranging from 28.7–37.5% (Fig. 2b). In CK treatment, Basidiomycota relative abundance increased with soil depth, while the SR treatment Basidiomycota revealed a decreasing trend. In CK treatment, at 0–10 cm soil profile, Basidiomycota decreased compared with SR treatment (*p* < 0.05) (Table S4). Moreover, in CK, the relative abundance of Glomeromycota significantly increased in 0–10 cm than 30–40 cm soil layer (*p* < 0.05). Compared to SR in 30–40 cm soil layers, Chytridiomycota relative abundance was 44.57 times more than in CK treatment. Compared to CK, SR application led to a significant increase of Cercozoa in 0–10 cm soil depth (*p* < 0.01).

### Community Structure, Variation, and Determinants

**Fig. 3:**
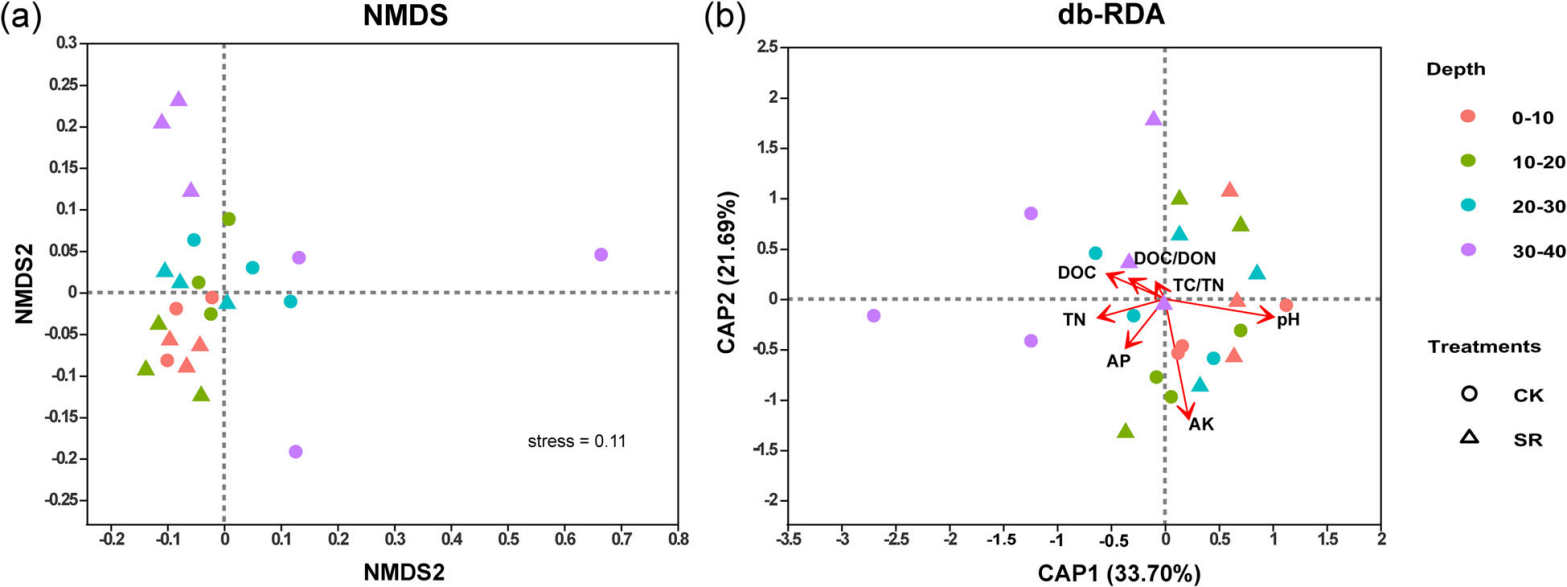
Analysis of (**a**) nonparametric multidimensional scaling (NMDS) and (**b**) distance-based Redundancy analysis (db-RDA) depicting fungal communities in different soil profiles (0–10, 10–20, 20–30, and 30–40-cm) . CK, control; SR, sugarcane straw retention

The NMDS based on unweighted UniFrac analysis at the OTU level revealed that fungal community similarity distance was influenced by both sugarcane straw retention and different soil depths (Fig. 3a). The analysis of similarities (ANOSIM) further confirmed significant differences between CK and SR (R = 0.66, *P* < 0.02). Additionally, the fungal community in topsoil (0–20 cm) varied from that subsoil (20–40 cm) (R = 0.54, *P* < 0.004) (Table S5). The analysis of VIF filtered the high Collinear factor TC and DON. The db-RDA was used at the OTU level to measure the effect of edaphic factors on fungal community composition, which demonstrated that pH (R^2^ = 0.46, *P* = 0.002), AK (R^2^ = 0.66, *P* = 0.001) and TN (R^2^ = 0.47, *P* = 0.03) were the major factors altering the fungal community structure (Fig. 3b).

### Fungal function prediction

**Fig. 4:**
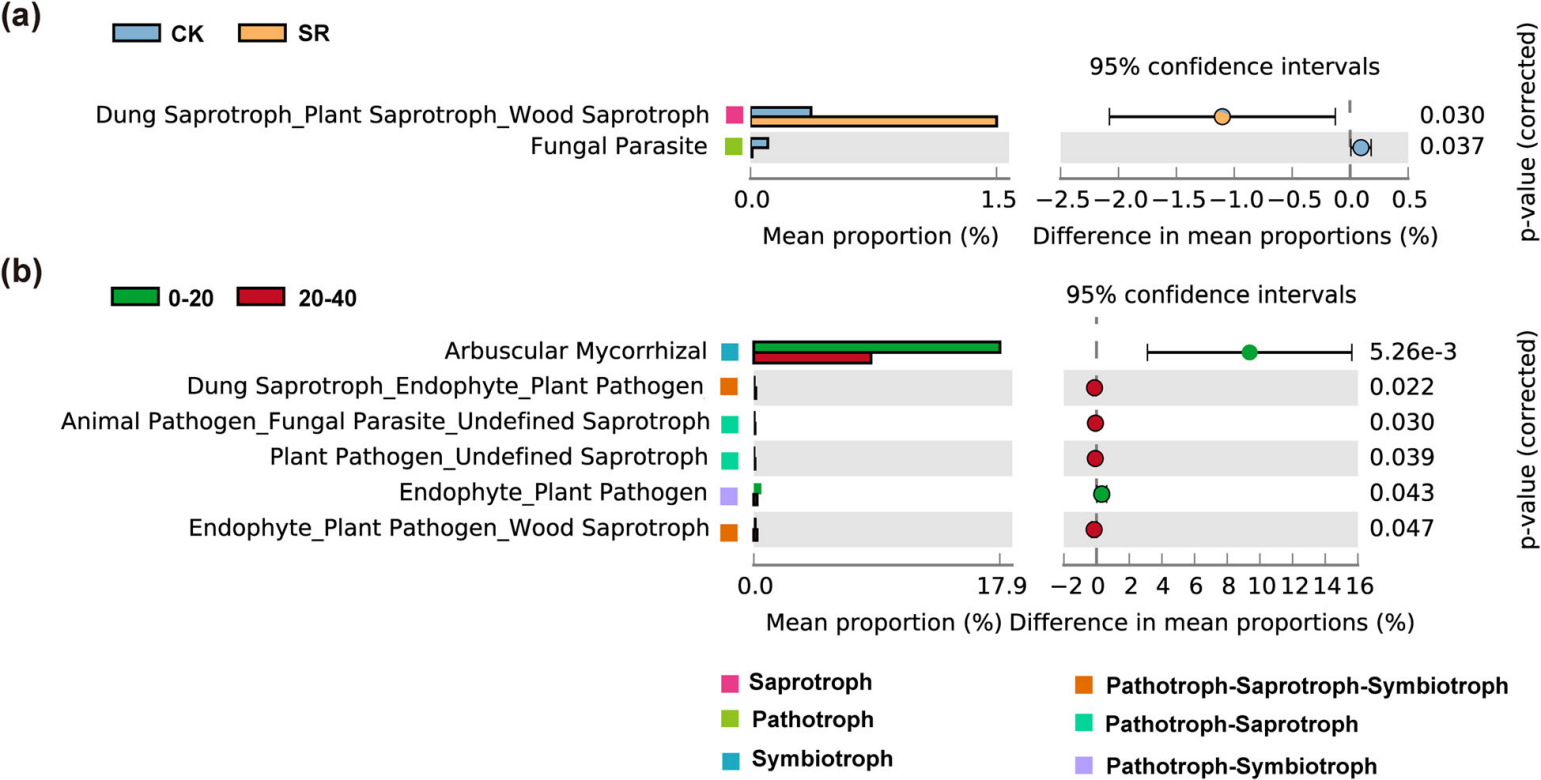
Extended error bar graphs indicate the significant difference of fungal functional guilds at level 2 (**a**) straw retention treatment and (**b**) in topsoil (0–20 cm) and subsoil (20–40 cm) (*p* < 0.05, average proportion, *n* = 3). The points depict variations between the “CK” and “SR”; between “0–20 cm” and “20–40 cm” depths, the values on the right-hand indicate the *p*-values obtained from the Welch t-test. **a** CK, control; SR, sugarcane straw retention

FUNGuild was used to analyze the metabolic pathways of soil fungi. Using an ecological guild and trophic mode, the fungi were classified in both treatments with different soil depths. Overall, a total of 29.14% of OTUs were classified as trophic modes with pathogenic, saprotrophic, and symbiotrophic, while the rest were not assigned. Saprotroph was the most observed fungi taxa in the samples. The relative abundance of saprotroph, and symbiotroph categories in CK treatment (18.90 and 6.57%, respectively) were lower than that in SR treatment (20.06 and 5.62%, respectively), while pathogen in CK (16.26%) was higher than in SR (7.60%) (Table S6). The proportion of the saprotroph category of dung saprotroph-plant saprotroph-wood saprotroph in SR treatment was significantly higher compared with CK treatment (*P* < 0.05) (Fig. 4a). The proportion of pathogen category of the fungal parasite in SR was lower than in the CK (*P* < 0.05). Different soil depths also varied the trophic modes. The relative abundance of pathogen and saprotrophic in topsoil depth (7.51 and 18.73%, respectively) were lower than subsoil (16.36 and 20.24%, respectively). Moreover, the relative abundance of symbiotroph in topsoil (8.04%) was higher than subsoil (4.16%). *Arbuscular mycorrhizal* (AM) and endophyte-plant pathogen relative abundance in topsoil were significantly higher compared to subsoil (*p* < 0.05) (Fig. 4b). On the other hand, dung saprotroph-endophyte-plant pathogen, animal pathogen-fungal parasite-undefined saprotroph, plant pathogen-undefined saprotroph, and endophyte-plant pathogen-wood saprotroph in topsoil diminished significantly compared to subsoil (*p* < 0.05). Compared to CK in 0–20 cm soil layers, the relative abundance of dung saprotroph-plant saprotroph-wood saprotroph and undefined saprotroph increased in SR, while the relative abundance of undefined saprotroph-wood saprotroph decreased in SR (Fig. S1). However, compared to CK in 20–40 cm soil layer, SR showed no significant difference.

### Fungal network

**Fig. 5:**
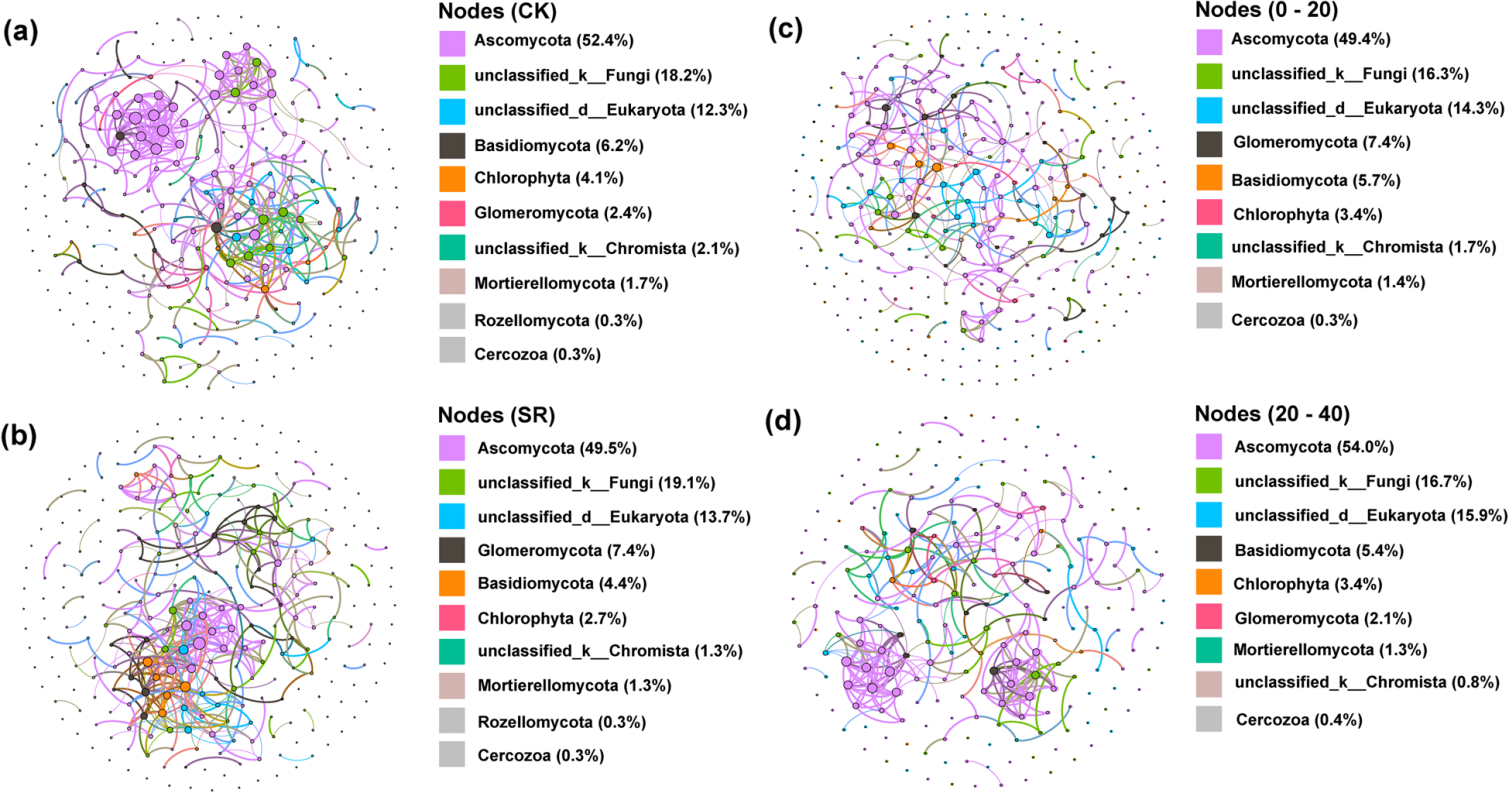
Co-occurrence networks with (**a**) non-straw retention, **b** straw retention, **c** topsoil (0–20 cm), and **d** subsoil (20–40 cm) of the fungal communities in the soil samples. CK, control; SR, sugarcane straw retention

Differences were observed between the two networks of fungal communities under straw retention and different soil profiles. The total nodes and edges in CK (292 and 410, respectively) were lower than in SR (299 and 412, respectively) (Fig. 5a, b). Meanwhile, negative edges and modularity in CK (17.32%, 0.97, respectively) were lower than SR (22.82%, 1.04) (Table S7). The relationships between nodes for each phylum pair were totaled to assess the potential interactions among the main phyla. The analysis of network topological properties revealed that SR showed a higher network average clustering coefficients (aveCC) than the CK treatment. The average path distance (GD) showed a decreasing trend from CK to SR treatment in fungal communities. In CK treatment, the nodes in the network were assigned to seven fungal phyla. At the phyla level, Ascomycota was widely distributed, representing 53% of all nodes. Nodes distribution was modularized, and clustered into 130 modules, with the majority of the nodes associated with Ascomycota. Based on betweenness centrality scores, the top four genera identified as keystone taxa were *Alternaria*, *unclassified-o-Hypocreales*, *unclassified-o-Branch06* and *Plectosphaerella*, which could be assorted into *Pleosporales*, *Hypocreales*, *Branch06* and *Glomerellales* orders respectively and also into a single Ascomycota phylum. The trophic mode of *Alternaria* and *Plectosphaerella* were associated with pathotroph-saprotroph-symbiotroph and pathotroph, respectively (Table S8). In SR treatment, the nodes in the network were assigned to seven fungal phyla. Similarly, the phylum Ascomycota was widely distributed, accounting for 50% of all nodes. After the distribution of nodes was modularized, nodes were grouped into 150 modules. The top four genera included four keystone taxa, *Trichoderma*, *Scutellinia*, *Plectosphaerella* and *Claroideoglomus*. The former three belonged to *Hypocreales*, *Pezizales*, *Glomerellales* orders and the same Ascomycota phylum, while the last one was associated with *Glomerales* order and Glomeromycota phylum. *Trichoderma* and *Scutellinia* were demonstrated with saprotroph, while *Plectosphaerella* and *Claroideoglomus* were demonstrated an association with pathotroph and symbiotroph, respectively. The total nodes and edges in topsoil (350 and 641, respectively) were higher than those in subsoil (239 and 511, respectively) (Fig. 5c, d), whereas average degree (avgK) 、aveCC and GD in topsoil (3.66, 0.70, and 6.43, respectively) were lower than those in subsoil (4.28, 0.71, and 8.05) (Table S7). Negative edges and modularity in subsoil (9.59%, 1.03, respectively) were lower than those in topsoil (14.20%, 1.61, respectively). Furthermore, in topsoil, the top four genera identified as keystone taxa were *Clitopilus* and *Auricularia* belonging to *Agaricales* and *Auriculariales* orders and Basidiomycota phylum, *Staphylotrichum* and *Pyrenochaetopsis* belonging to *Sordariales* and *Pleosporales* orders and Ascomycota phylum. *Clitopilus*, *Auricularia* and *Staphylotrichum* were saprotroph, *Pyrenochaetopsis* was pathotroph-saprotroph-symbiotroph (Table S8). In the subsoil, keystone taxa were *Abortiporus* belonging to *Polyporales* order and the phylum of Basidiomycota, and *Trebouxia* was related to *Trebouxiales* order and Chlorophyta phylum. Besides, *unclassified-o-Branch06* and *unclassified-f-Didymellaceae* demonstrated an association with the phylum of Ascomycota, which belongs to *Branch06* and *Pleosporales* orders. And *Abortiporus* was associated with saprotroph. Compared to CK at 0–20 cm, the edges and modularity increased in SR, while positive edges decreased in SR (Fig. S2a; b) (Table S7). Compared to CK at 20–40 cm, the edges and avgK decreased in SR (Fig. S2c; d) (Table S7).

### Pearson’s correlation between soil attributes and relative abundance of soil fungal taxa

**Fig. 6:**
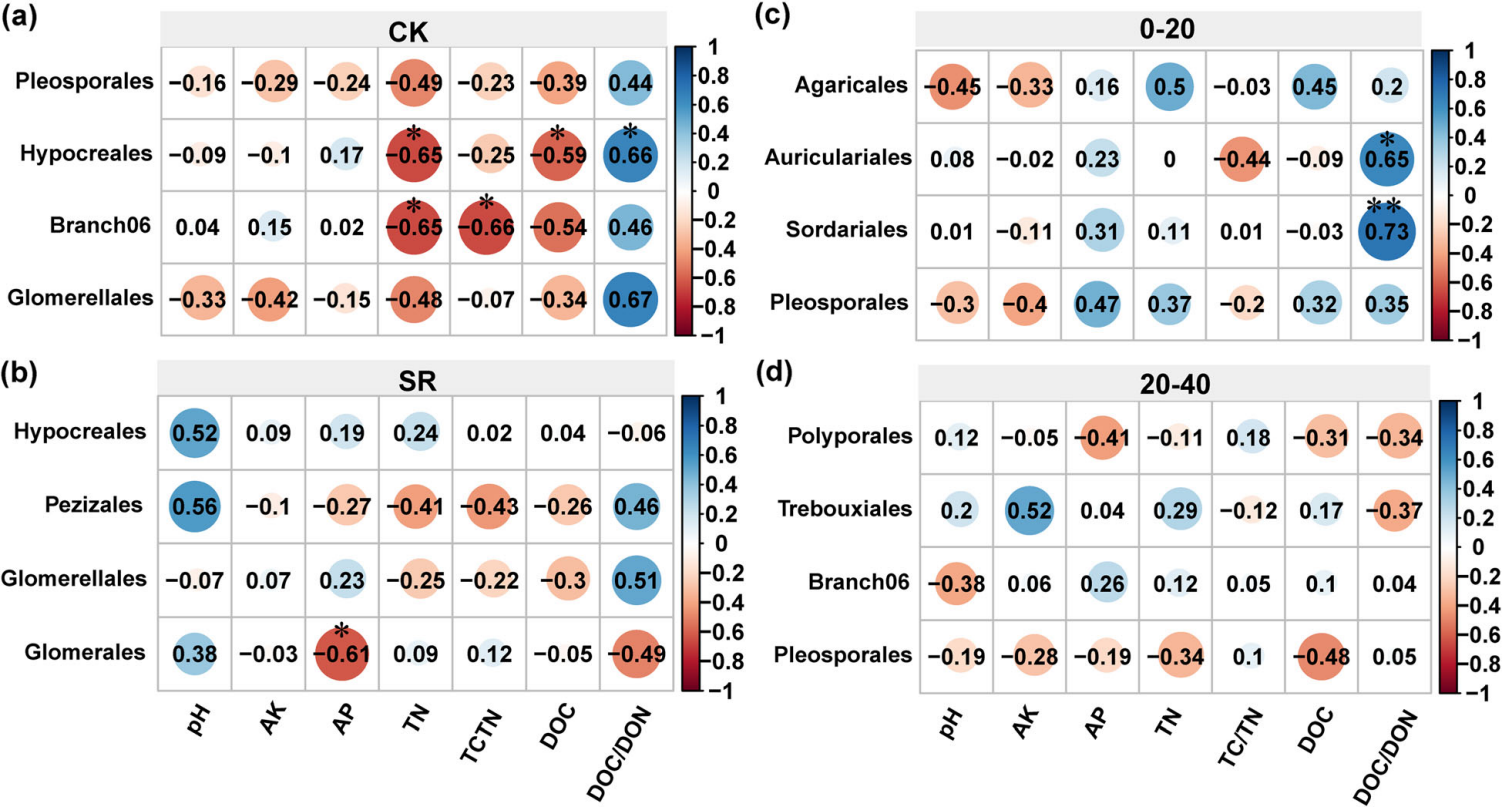
Pearson’s correlation coefficients of soil physiochemical properties and the keystone taxa of a network (Order), **a** non-straw retention, **b** straw retention, and **c** topsoil (0–20 cm), and **d** subsoil (20–40 cm) depths. The heatmap cells marked by “*” or “**” are statistically significant: * *p* < 0.05 and ** *p* < 0.01

Pearson’s correlation coefficients indicated that the keystone taxa of a network (Order) correlated with multiple soil properties. The keystone taxa were from network (order) in both CK and SR treatments, as well as different soil depths (topsoil and subsoil). In CK treatment, soil TN and DOC were significant negative correlation with *Hypocreales* (*p* < 0.05), however, DOC/DON revealed positive correlation as well (Fig. 6a). *Hypocreales* revealed an association with pathotroph - saprotroph - symbiotroph trophic mode (Table S9). TC and TC/TN were negatively and significantly related to *Branch06* (*p* < 0.05). In SR treatment, AP was negatively and significantly connected with *Glomerales* (*p* < 0.05) (Fig. 6b), which was associated with symbiotroph. In the topsoil DOC/DON revealed a positive and significant relationship with *Auriculariales* and *Sordariales* (*p* < 0.05 and *p* < 0.01, respectively), which were saprotroph (Fig. 6c). Subsoil nutrient revealed no significant association with keystone taxa (Fig. 6d). The keystone taxa were from the network (genus), which were correlated with C and N cycle (Fig. S3). Soil pH was significantly positively correlated with *unclassified-Hypocreales. Unclassified-o-Branch06* and *Claroideoglomus* were negatively correlated with soil C and N cycle. However, *Clitopilus* and *Trebouxia* were positively correlated with soil C and N cycle. Furthermore, *Plectosphaerella* was negatively correlated with TC/TN in CK, howe*ver, Plectosphaerella* was positively correlated with DOC/DON in SR.

## Discussion

Research shown that straw retention can alter soil microbial distribution throughout the soil profile [40]. Similarly, in this study showed that straw retention improved fungal abundance, especially in topsoil, while it decreased exponentially with increasing soil depth. The previous study has shown that fungi dominated litter-C decomposition, and fungal community composition varied within different soil profiles and controlled resource availability [41]. It is well documented that different organic materials, especially wheat straw, farm manure [42], and cow manure compost, change microbial biomass and agricultural land activity [2]. A similar study showed that straw retention positively impacts cucumber seedlings growth by increasing soil microbial biomass and changing soil microbial community structure [43].

Increased biodiversity can promote the stability of ecosystems and enhance the mix of basic microbial functions and activities [44]. Alpha fungus diversity decreased with increasing soil depth [45], which is supported by our results that straw retention decreased fungal diversity at a soil depth of 0–30 cm than 0–10 cm. Compared with CK, Fungal richness showed no obvious change in response to straw retention, while soil depth was the main driving force to change fungal diversity, which is consistent with previous studies [41, 46, 47].

Fungi play a key role in decomposing recalcitrant substrates [48, 49], and their abundance changed under the combined effect of treatment and soil depth*.* Ascomycota plays a key role in the decomposition of organic substrates [30, 32, 50] and is found to be the main phyla of fungi. Furthermore, SR improved the relative abundance of Ascomycota at different soil depths. Straw retention significantly improved Basidiomycota abundance, which is consistent with previous findings [51]. As an important decomposer, Basidiomycota produces enzymes (e.g, peroxide) to degrade recalcitrant plant compounds, such as cellulose and lignin [50]. A previous study showed that straw retention could increase carton content and cellulase activity [18].

Funguild analysis revealed that straw retention not only altered saprotroph (dung saprotroph-plant saprotroph-wood saprotroph) but also suppressed pathogenic (fungal parasites) (Fig. 4a). A similar phenomenon was detected in the composting of Chinese herb residues [52]. Many studies have confirmed that saprotrophs are involved in the decomposition process [53], and pathogenic fungi commonly acquire nutrients for invading host cells, so they are known to pose a threat to other fungal community members [54]. Thus, the result showed that straw retention can improve soil nutrient cycles and health. Many investigations indicated that between plant roots and a diverse array of mutualistic endophytic symbionts enhance crop quality, health, and soil nutrition [55, 56]. For example, AM can protect plant root and improve plant nutrient absorption capacity [57, 58]. Meanwhile, its variability depends on soil depth [59], same as our result. Pathogens in subsoil were higher than topsoil, which harms other fungal community members This finding corresponds to a previous study, in which the relative abundance of soil pathogenic fungi increased with increasing soil depth [45].

The ecological network of biological communities has been extensively studied in animal and plant ecology and has recently received microbial ecology attention. However, current research provides insights into the effect of straw retention on changes in fungal communities, with a focus on soil depth. Our findings revealed that the fungal community network in straw retention and topsoil revealed negative edges and modularity network (Table S7). If the degree of modularity of the two networks exceeds 0.4, it indicates that these networks are modular [60]. Many studies have shown that the existence of modularity and negative interactions enhance the stability of disturbed networks [61–64]. The AveCC of SR treatment and topsoil were higher than CK and subsoil, showing that there were more potential connections and small-world behavior. In a small-world network, more OTUs could be available to all other OTUs via a comparatively short path [65]. On the contrary, the more connected the network is, the more it can contribute to effective and efficient carbon utilization [65]. Betweennes centrality scores indicates how often a node is found on the shortest path between two nodes in the network to connect it to each other, the higher number, the more connected they are. Network analysis showed that Ascomycota was identified as the main phylum for straw retention and soil depth, indicating that they played an important role in maintaining the function and structure of the ecological community. Keystone taxa were correlated with the C and N cycle in the CK and SR treatments. In the CK treatment, the number of *Pleosporales* was higher in the CK than SR. Species of *Pleosporales* occurred in various habitats, that can be epiphytes, endophytes or parasites of living leaves or stems, hyperparasites on fungi or insects, lichenized, or are saprobes of dead plant stems, leaves or bark) [66]. While *Hypocreales* order was present in SR treatment. Sordariomycetes are soft-rot fungi, which are well known to effectively decompose organic substrates such as cellulose, cellobiose and lignin [67].

These results showed that the keystone taxa were involved in various carbon and nitrogen substrates, such as TN, TC/TN, DOC, and DOC/DON, P cycle and AP utilization. This finding is consistent with previous studies documenting that different soils can support different fungal flora [68]. *Hypocreales*, which belongs to the patotroph, were also reported to be negatively correlated with TN and DOC [69]. A previous study indicated that environmental factors, such as TN, DOC, and DOC/DON were unfavorable conditions for *Hypocreales*, and also postulated that excess nutrients decreased chlamydospore production [70]. In a related study, *Glomerales*, which belongs to *arbuscular mycorrhiza fungi* (AMF) [71], was associated with a high amount of available P, which in turn boosted plant growth [58]. Additionally, *Auriculariales* and *Sordariales* are generally considered saprophytic fungi [72], which stimulate the decomposition of organic substrates by saprotrophic fungi [73].

## Conclusions

This study, we demonstrated that the fungal community composition, function, and co-occurrence pattern changed significantly in response to straw retention throughout the soil profile. The straw retention increased the diversity and abundance of fungi in 0–10 cm soil depth. Both straw retention and topsoil had a decreasing effect on the abundance of pathogens. Straw retention and depth of soil influenced the keystone taxa. Overall, these findings enhance our understanding of fungal metabolic functions and networks under straw retention in different soil profiles.

## Materials and methods

### Field site and experiment design

Short-term (14 months) experiment using sugarcane straw retention started in March 2017 at the Sugarcane Research Center of Fujian Agriculture and Forestry University, Fuzhou, Fujian Province, China (latitude: 26°05′9.60″ N; longitude: 119°14′3.60″ E) in the fallow ecosystem. The site has a clay loam texture, an annual average temperature of 20 °C and rainfall of 1369 mm with a subtropical monsoon climate. The data of soil biological properties showed in our published research [18]. The sugarcane straw used in this study was collected from an adjacent sugarcane field, and crushed into small pieces. Two treatments, e.g., (i) control (CK), moldboard plow at 40 cm depth in the fallow field without sugarcane straw retention; and (ii) sugarcane straw retention (SR), moldboard plow at 40 cm depth in a fallow field with 30 t ha^− 1^ of sugarcane straw retention were laid out, with three replicates. After SR, all field plots remained unplanted for1 year without any fertilization.

In May 2018, five sampling points were randomly taken from each plot and homogenized as one mixed sample. Based on the soil profile, soil samples were collected at depth 0–10, 10–20, 20–30, and 30–40 cm. Finally, a total of 24 soil samples were obtained from the experiment site and taken to the laboratory on ice. Samples were mixed thoroughly and sieved (2 mm), and divided into parts. A portion of the fresh soil was air-dried to measure soil physiochemical properties. Furthermore, approximately 50 g of soil was packed into a sterile bag, and stored at − 80 °C.

### DNA extraction

The total genomic DNA was extracted from 0.5 g newly collected soil with three replicates using the Fast DNA™ Spin Kit (MP Biomedicals, LLC, Santa Ana, USA) according to the manufacturer’s instructions. DNA concentration and quality were measured by calculating their absorbance (A260 and 280 nm) using BioTek Synergy H1 Hybrid Multi-Mode Microplate Reader (BioTek, USA). DNA was diluted with sterile water to a final concentration of 20 ng μL^− 1^ for qRT-PCR. The integrity of the DNA extracts was ensured by electrophoresis and was stored at − 80 °C awaiting sequencing.

### qRT-PCR

The qRT-PCR method was employed to quantify soil fungi abundance using the primer set ITS1-F (5′-CTTGGTCATTTAGAGGAAGTAA-3′) [74] and ITS4-R (5′-TCCTCCGCTTA- TTGATATGC-3′) [75] and SYBR Green detection (FastFire qPCR PreMix, TianGen Biotech, China). The standard for calculating the *ITS rRNA* gene quantity was developed from a clone with the correct insert. A plasmid DNA was generated from the clone using the pEASY®-T1 Simple Cloning Kit (Transgene, China). The R^2^ of the standard curve was > 0.99. The qRT-PCR reactions were carried out using each extracted DNA sample.

### Illumina MiSeq sequencing

The amplification of the hypervariable ITS3–4 region of the *ITS rRNA* gene was carried out using fungal primers set ITS3F (GCATCGATGAAGAACGCAGC) and ITS4R (TCCTCCGCTTATTGATATGC) [76]. The PCR reactions were carried out in a 50 μL mixture with 1 mM dNTPs, 1× PCR buffer, 1 U Platinum Taq, 5 μM per primer, and 10 ng of template DNA. The PCR amplification included an initial denaturation at 94 °C for 3 min, denaturation (5 cycles at 94 °C) for 30 s, annealing at 45 °C for 20 s, extension at 65 °C for 30 s, denaturation (20 cycles at 94 °C) for 20 s, annealing at 55 °C for 20 s, extension at 72 °C for 30 s and a final extension at 72 °C for 5 min. After purification and quantification, the PCR product of the ITS3–4 region of the *ITS rRNA* gene was determined by pyrosequencing using an Illumina MiSeq sequencer (Sangon Biotech Shanghai Co., Ltd., China) [18, 77].

### Processing and analyzing of sequencing data

Both QIIME (version 1.17) software package and UPARSE software (version 7.1) was used to process raw sequences [78]. Sequences quality score < 20, length < 250 bp, or reads containing ambiguous characters were removed. After overlapped, sequences more than 10 bp in size were assembled based on their sequence overlaps, unassembled sequence reads were eliminated, and sequences with ≥97% similarity were clustered into operational taxonomic units (OTUs), while chimeric sequences were identified and eliminated through the UCHIME method [79]. For each OTU, representative sequences were chosen for each OUT. A Ribosomal Database Project (RDP) classifier [80] was adopted to annotate the taxonomic information for each representative sequence. The species richness (ACE and Chao1 indexes) [81, 82], number of observed OTUs, and diversity (Shannon index) [83] were used to calculate fungal abundance, diversity and communities in each soil sample using the Mothur pipeline [84]. Nonmetric multidimensional scale (NMDS) analysis was carried out to assess the variation in fungal community structure across the different soil layers [19, 85]. Environmental factors were filtered with VIF (Variance Inflation Factor), factors greater than 10 were removed multiple times until the VIF values corresponding to the selected. Analysis of similarity (ANOSIM) was conducted to estimate the dissimilarity in the fungal community structures with treatments (CK and SR, 0–20 cm and 20–40 cm depths) using unweighted UniFrac dissimilarities. Furthermore, a distance-based redundancy analysis (db-RDA) was also used to examine the impact of soil physiochemical properties (Table S1) on fungal community composition among the different soil layers [18]. Pearson’s correlation analysis was separately determined for treatments (CK, SR and topsoil, subsoil) to investigate the interaction among soil physiochemical properties and fungal order taxa, using R-software 3.5.2. The test data were analyzed using ANOVA by IBM SPSS Statistics software, and the difference between the mean values of each treatment was compared by Tukey’s procedure at a 5% level [19].

### FUNGuild analysis

Fungi community function was investigated using FUNGuild to identify the functional groups (guilds) in the straw retention experiment. Fungi functional guild of was carried out using FUNGuild v1.0 [26], which taxonomically parsed fungal OTUs by examining the ecological guild of sequencing databases. Three trophic modes, for example, saprotrophs pathotrophs, and symbiotrophs are widely well-defined types in the fungal community ecology as they determine the specific fungi feeding habits. Twelve guilds related to these trophic modes were categorized. The Guilds that were “highly probable” and “probable” in the assignments were selected for not over-interpreting their data ecologically. OTUs of each sample that did not match taxa in the database were categorized as “unassigned”.

### Network analysis

Dynamic networks have great visualizations benefits, which depict ideas and concepts not immediately visible in a sociogram static. To minimize the complexity, only abundant OTUs with a proportion of total reads over 0.01% were retained in the OTUs table. OTUs table was then analyzed using R software 3.5.2 with the packages “psych” for the correlation matrix. The correlation matrix table result was submitted in Gephi. Gephi, is an interactive visualization and exploration platform used for complex systems graphs and many networks [86, 87]. Betweenness centrality (BC) was employed to determine the importance of the network structure, and high BC scores were very essential in sustaining the connectivity of an ecological network and matched them with key keystone species [88, 89]. Modularity analysis determines how well a network may be separated into smaller clusters, or modules [89], and can be very important in identifying fungi community structure. High modularity depicts a network higher rate of intra-module edges relative to inter-module ones [90]. Gephi uses a modularity algorithm called the Louvain method, developed by Blondel and colleagues (2008) to find communities in the network [91].

## Supplementary Information

**Additional file 1: Table S1.** Soil physiochemical properties at different depths. **Table S2.** The copies number of *ITS rRNA* gene at 0–40-cm depths. **Table S3.** Distribution of the number of tags across the soil samples. **Table S4.** Relative abundances of the fungal phyla at different depths. **Table S5.** The ANOSIM result of pairwise comparison. **Table S6.** The Composition proportion of fungal functional groups (guilds) inferred by FUNGuild. **Table S7.** Properties of fungal co-occurrence networks. **Table S8.** Variation in fungal functional group compositions of fungal communities at the genus level. **Table S9.** Variation in fungal functional group compositions of fungal communities at the order level. **Figure S1.** Extended error bar graphs indicate the significant difference of fungal functional guilds with control and straw retention treatment in topsoil (0–20 cm) (*p* < 0.05, average proportion, *n* = 3). The points depict variations between the “CK0–20” and “SR0–20”, the values on the right-hand indicate the *p*-values obtained from the Welch t-test. CK, control; SR, sugarcane straw retention. **Figure S2.** Co-occurrence networks with (a) CK0–20, (b) SR0–20, (c) CK20–40, and (d) SR20–40 of the fungal communities in the soil samples. CK, control; SR, sugarcane straw retention. **Figure S3**. Pearson’s correlation coefficients of soil physiochemical properties and the keystone taxa of a network (genus), (a) non-straw retention, (b) straw retention, and (c) topsoil (0–20 cm), and (d) subsoil (20–40 cm) depths. The heatmap cells marked by “*” or “**” are statistically significant: * *p* < 0.05 and ** *p* < 0.01.

## Data Availability

All datasets are presented in the main text and the additional file. The raw sequence data on *ITS rDNA* gene amplicons have been submitted to the NCBI Sequence Read Archive (SRA) database (Accession Number: SRP289388). The dataset analyzed during the current study is available from the corresponding author on reasonable request. [https://dataview.ncbi.nlm.nih.gov/object/PRJNA671590].

## Abbreviations

CK: Control
SR: Sugarcane straw retention
HTS: High throughput sequencing
ANOSIM: Analysis of similarities
AM: Arbuscular mycorrhizal
avgK: Average degree
aveCC: Average clustering coefficients
NMDS: Nonmetric multidimensional scale
RDP: Ribosomal Database Project
VIF: Variance Inflation Factor
db-RDA: distance-based redundancy analysis
OTU: Operational Taxonomic Units
rRNA: Ribosomal RNA
PCR: Polymerase Chain Reaction
ANOVA: Analysis of Variance
