# Supplementary material for: Straw retention efficiently improves fungal communities and functions in the fallow ecosystem
Source: BMC Microbiol. 2021 Feb 17;21:52. doi: 10.1186/s12866-021-02115-3 (PMC7890633; doi:10.1186/s12866-021-02115-3)
Supplement: Supplementary file 1 — Additional file 1: Table S1. Soil physiochemical properties at different depths. Table S2. The copies number of ITS rRNA gene at 0–40-cm depths. Table S3. Distribution of the number of tags across the soil samples. Table S4. Relative abundances of the fungal phyla at different depths. Table S5. The ANOSIM result of pairwise comparison. Table S6. The Composition proportion of fungal functional groups (guilds) inferred by FUNGuild. Table S7. Properties of fungal co-occurrence networks. Table S8. Variation in fungal functional group compositions of fungal communities at the genus level. Table S9. Variation in fungal functional group compositions of fungal communities at the order level. Figure S1. Extended error bar graphs indicate the significant difference of fungal functional guilds with control and straw retention treatment in topsoil (0–20 cm) (p < 0.05, average proportion, n = 3). The points depict variations between the “CK0–20” and “SR0–20”, the values on the right-hand indicate the p-values obtained from the Welch t-test. CK, control; SR, sugarcane straw retention. Figure S2. Co-occurrence networks with (a) CK0–20, (b) SR0–20, (c) CK20–40, and (d) SR20–40 of the fungal communities in the soil samples. CK, control; SR, sugarcane straw retention. Figure S3. Pearson’s correlation coefficients of soil physiochemical properties and the keystone taxa of a network (genus), (a) non-straw retention, (b) straw retention, and (c) topsoil (0–20 cm), and (d) subsoil (20–40 cm) depths. The heatmap cells marked by “*” or “**” are statistically significant: * p < 0.05 and ** p < 0.01. [file 12866_2021_2115_MOESM1_ESM.docx]

**Straw Retention Efficiently Improves Fungal Communities and Functions in the Fallow Ecosystem**

Caifang Zhang ^1,2^, Zhaoli Lin ^1,2^, Youxiong Que ^1,2^, Nyumah Fallah ^1,2^, Muhammad Tayyab ^1,2^, Shiyan Li ^1,2^, Jun Luo ^1,2^, Zichu Zhang^3^, Ahmad Yusuf Abubakar ^1,2^and Hua Zhang ^1,2*^

^1^ *Key Laboratory of Sugarcane Biology and Genetic Breeding, Ministry of Agriculture, Fujian Agriculture and Forestry University, Fuzhou 350002, China. 000xm19027@fafu.edu.cn (C.Z.); 000l812060@fafu.edu.cn (Z.L.); [000q010088@fafu.edu.cn (Y.Q.);](mailto:queyouxiong@126.com;) 1181901001@fafu.edu.cn (N.F.); tayyab@fafu.edu.cn (M.T.); [lishiyan@fafu.edu.cn](mailto:lishiyan@fafu.edu.cn) (S.L.); 000q010023@fafu.edu.cn (J.L.); 1171902001@fafu.edu.cn (A.Y.A.).*

^2^ *College of Agriculture, Fujian Agriculture and Forestry University, Fuzhou 350002, China*

^3^ *Fuzhou No.8 High School, Fuzhou 350000, China. [zichu.zhang@fz.lyndoninstitute.org.cn](mailto:zichu.zhang@fz.lyndoninstitute.org.cn) (Z.Z.).*

**^*^Corresponding author**. *E-mail address*: [000q010025@fafu.edu.cn](mailto:000q010025@fafu.edu.cn) (H.Z.)

*Telephone number*: +86-1869-570-2152

**Supplementary Materials**

| **Table S1** Soil physiochemical properties at different depths | | | | | | | | | | |
| --- | --- | --- | --- | --- | --- | --- | --- | --- | --- | --- |
| **Treatments** | **Depth** | **DOC** | **DON** | **TC** | **TN** | **C/N** | **DOC/DON** | **pH** | **AK** | **AP** |
|  |  | **mg/kg** | **mg/kg** | **g/kg** | **g/kg** |  |  |  | **mg/kg** | **mg/kg** |
| CK | 0-10 | 41.88±1.88b | 13.57±1.55bc | 7.83±0.17ab | 0.82±0.02ab | 9.57±0.04abc | 3.18±0.44abc | 6.49±0.02c | 93.51±4.36de | 10.28±0.20a |
| CK | 10-20 | 33.82±0.83d | 9.60±0.32c | 6.59±0.23c | 0.69±0.01c | 9.57±0.20abc | 3.53±0.08abc | 6.57±0.01b | 105.40±0.61abcd | 9.98±1.13a |
| CK | 20-30 | 33.89±0.46d | 11.42±0.76bc | 5.58±0.23e | 0.62±0.02de | 9.04±0.09c | 2.99±0.18abc | 6.56±0.03b | 98.84±9.16bcd | 9.83±0.50a |
| CK | 30-40 | 33.43±0.89d | 9.48±1.75c | 5.51±0.15e | 0.59±0.02e | 9.35±0.17bc | 3.80±0.74ab | 6.50±0.02c | 85.91±2.48e | 9.53±1.34a |
| SR | 0-10 | 46.66±1.02a | 23.88±6.14a | 8.40±0.23a | 0.86±0.02a | 9.71±0.13ab | 2.35±0.76bc | 6.64±0.01a | 105.97±2.04abc | 9.38±0.16a |
| SR | 10-20 | 38.09±0.46c | 18.10±2.31ab | 7.60±0.21b | 0.76±0.02b | 9.99±0.27a | 2.19±0.35c | 6.61±0.01ab | 111.83±2.68a | 9.15±0.56a |
| SR | 20-30 | 34.18±1.74d | 12.30±1.63bc | 6.28±0.15cd | 0.68±0.03cd | 9.27±0.21bc | 2.94±0.60abc | 6.58±0.01b | 110.63±0.98ab | 9.36±0.29a |
| SR | 30-40 | 34.23±1.09d | 8.35±0.76c | 5.91±0.10de | 0.64±0.03cde | 9.31±0.26bc | 4.15±0.28a | 6.57±0.02b | 94.30±2.96cde | 9.07±0.84a |
| Soil physiochemical properties at 0–10, 10–20, 20–30, and 30–40 cm depths in the sugarcane straw retention (SR) treatment compared to the control (CK). Different lowercase letters show significant differences between treatments (Tukey test, n = 3, p < 0.05). DOC, dissolved organic carbon; DON, dissolved organic nitrogen; TC, total soil carbon; TN, total nitrogen; C/N, carbon/nitrogen ratio; DOC/DON, dissolved organic carbon/dissolved organic nitrogen ratio; pH, potential hydrogen; AK, available potassium; AP, available phosphorus. | | | | | | | | | | |

| **Table S2** The copies number of *ITS rRNA* gene at 0–40-cm depths   \| **Treatments (A)** \| **Depth (B)** \| **ITS rRNA Gene copies number** \| \| --- \| --- \| --- \| \| CK \| 0-10 \| 5.80E+07 (± 1.34E+07 ) b \| \| CK \| 10-20 \| 2.75E+07 (± 5.99E+06 ) c \| \| CK \| 20-30 \| 1.47E+07 (± 4.24E+06 ) c \| \| CK \| 30-40 \| 6.48E+06 (± 2.33E+06 ) c \| \| SR \| 0-10 \| 8.52E+07 (± 1.74E+07 ) a \| \| SR \| 10-20 \| 3.14E+07 (± 6.55E+06 ) c \| \| SR \| 20-30 \| 1.52E+07 (± 1.54E+06 ) c \| \| SR \| 30-40 \| 1.02E+07 (± 4.93E+05 ) c \| \|  \| A \| 2.15 \| \|  \| B \| 22.08** \| \|  \| A×B \| 1.05 \|   Distribution of the number of tags across the soil samples. CK: non-straw retention treatment, SR:sugarcane straw retention treatment. 0-10, 10-20, 20-30 and 30-40 represent soil samples at 0-10, 10-20, 20-30 and 30-40 cm depths. The result was used SPSS 20 statistics for one-way analysis of variance (ANOVA) for the Tukey’s test. The various letters represent the level of significance of the sample (p < 0.05). ** represent the level of significance at 0.01.  **Table S3**. Distribution of the number of tags across the soil samples | | | | | |
| --- | --- | --- | --- | --- | --- | --- | --- | --- | --- | --- | --- | --- | --- | --- | --- | --- | --- | --- | --- | --- | --- | --- | --- | --- | --- | --- | --- | --- | --- | --- | --- | --- | --- | --- | --- | --- | --- | --- | --- | --- | --- |
| **Group** | **Sample** | **Seq_num** | **Organelle_num** | **Chimeras_num** | **Filtered_num** |
| CK0-10 | CK0-10-1 | 39360 | 0 | 356 | 39004 |
| CK0-10 | CK0-10-2 | 45573 | 0 | 127 | 45446 |
| CK0-10 | CK0-10-3 | 34249 | 0 | 337 | 33912 |
| CK10-20 | CK10-20-1 | 58385 | 0 | 681 | 57704 |
| CK10-20 | CK10-20-2 | 42840 | 0 | 230 | 42610 |
| CK10-20 | CK10-20-3 | 31654 | 0 | 97 | 31557 |
| CK20-30 | CK20-30-1 | 42151 | 0 | 287 | 41864 |
| CK20-30 | CK20-30-2 | 58130 | 0 | 660 | 57470 |
| CK20-30 | CK20-30-3 | 55755 | 0 | 242 | 55513 |
| CK30-40 | CK30-40-1 | 58370 | 0 | 1101 | 57269 |
| CK30-40 | CK30-40-2 | 48009 | 0 | 495 | 47514 |
| CK30-40 | CK30-40-3 | 56712 | 0 | 351 | 56361 |
| SR0-10 | SR0-10-1 | 42186 | 0 | 583 | 41603 |
| SR0-10 | SR0-10-2 | 35215 | 0 | 278 | 34937 |
| SR0-10 | SR0-10-3 | 28719 | 0 | 201 | 28518 |
| SR10-20 | SR10-20-1 | 27914 | 0 | 142 | 27772 |
| SR10-20 | SR10-20-2 | 37821 | 0 | 325 | 37496 |
| SR10-20 | SR10-20-3 | 28063 | 0 | 46 | 28017 |
| SR20-30 | SR20-30-1 | 17722 | 1 | 436 | 17285 |
| SR20-30 | SR20-30-2 | 35079 | 0 | 493 | 34586 |
| SR20-30 | SR20-30-3 | 28884 | 0 | 258 | 28626 |
| SR30-40 | SR30-40-1 | 35911 | 0 | 200 | 35711 |
| SR30-40 | SR30-40-2 | 45090 | 0 | 144 | 44946 |
| SR30-40 | SR30-40-3 | 34643 | 0 | 112 | 34531 |

Distribution of the number of tags across the soil samples. CK 0-10, CK 10-20, CK 20-30 and CK 30-40 represent soil samples derived from non-straw retention treatment at 0-10, 10-20, 20-30 and 30-40 cm depths. SR 0-10, SR 10-20, SR 20-30 and SR 30-40 represent soil samples taken from the sugarcane straw retention treatment at depths of 0-10, 10-20, 20-30 and 30-40 cm.

**Table S4** Relative abundances of the fungal phyla at different depths

| **Treatment** | **Ascomycota** | **Basidiomycota** | **Cercozoa** | **Chytridiomycota** | **Glomeromycota** | **Mortierellomycota** |
| --- | --- | --- | --- | --- | --- | --- |
| CK0-10 | 23.44±1.05b | 1.43±0.15c | 0.44±0.07cde | 0.04±0.02b | 3.74±1.56a | 0.73±0.11bc |
| CK10-20 | 30.74±0.40b | 1.79±0.29bc | 0.76±0.06abc | 0.19±0.05b | 3.20±0.42ab | 2.15±0.73ab |
| CK20-30 | 30.76±5.00b | 2.08±0.16bc | 0.80±0.17ab | 0.18±0.10b | 1.69±0.05ab | 3.01±1.38a |
| CK30-40 | 50.00±6.08a | 3.14±0.57bc | 0.21±0.09e | 3.42±1.90a | 1.00±0.45b | 1.31±0.27abc |
| SR0-10 | 34.42±10.24b | 9.76±1.52a | 1.01±0.10a | 0.19±0.10b | 3.65±0.65ab | 0.93±0.11bc |
| SR10-20 | 37.51±4.71ab | 3.75±0.35b | 0.86±0.13ab | 0.15±0.07b | 4.00±0.79a | 1.58±0.12abc |
| SR20-30 | 35.96±3.72ab | 2.21±0.73bc | 0.61±0.11bcd | 0.10±0.04b | 2.29±1.27ab | 1.97±0.27abc |
| SR30-40 | 28.70±3.02b | 1.57±0.30c | 0.40±0.15de | 0.08±0.05b | 1.65±0.99ab | 0.37±0.26c |

Relative abundances of the fungal phyla at different depths in control (CK) treatments and) the sugarcane straw retention (SR). CK, control; and SR, sugarcane straw retention. The various letters represent the level of significance of the sample (p < 0.05).

| \| **Table S5** The ANOSIM result of pairwise comparison \| \| \| \| --- \| --- \| --- \| \| **Pairwise comparison** \| **R^2^** \| **P** \| \| CK vs. SR \| 0.66 \| 0.02 \| \| 0-10 vs. 10-20 \| 0.11 \| 0.31 \| \| 0-10 vs. 20-30 \| 0.41 \| 0.22 \| \| 0-10 vs. 30-40 \| 0.22 \| 0.02 \| \| 10-20 vs. 20-30 \| 0.04 \| 0.39 \| \| 10-20 vs. 30-40 \| -0.11 \| 0.60 \| \| 0-10 vs. 10-30 \| 0.04 \| 0.41 \| \| 0-20 vs. 20-40 \| 0.54 \| 0.004 \| \| 0-30 vs. 30-40 \| 0.63 \| 0.12 \| \| 0-10, 10-20, 20-30 and 30-40 cm represent depths of soil. The ANOSIM analysis of sample grouping information, random replacement test 999 times using R language. \| \| \| \| \|   **Table S6** The Composition proportion of fungal functional groups (guilds) inferred by FUNGuild | | | | | |
| --- | --- | --- | --- | --- | --- | --- | --- | --- | --- | --- | --- | --- | --- | --- | --- | --- | --- | --- | --- | --- | --- | --- | --- | --- | --- | --- | --- | --- | --- | --- | --- | --- | --- | --- | --- | --- | --- | --- | --- | --- | --- |
| **Trophic Mode** | **Guild** | **CK (%)** | **SR (%)** | **0-20 (%)** | **20-40 (%)** |
| Pathogen | Animal Pathogen | 0.80 | 0.60 | 0.87 | 0.53 |
|  | Plant Pathogen | 15.46 | 7.00 | 6.64 | 15.83 |
| Saprotroph | Dung Saprotroph | 1.82 | 1.80 | 2.75 | 0.88 |
|  | Plant Saprotroph | 0.00 | 0.01 | 0.01 | 0.01 |
|  | Soil Saprotroph | 0.06 | 0.08 | 0.03 | 0.11 |
|  | Wood Saprotroph | 16.18 | 17.50 | 15.07 | 18.61 |
|  | Undefined Saprotroph | 0.84 | 0.67 | 0.87 | 0.63 |
| Symbiotroph | Arbuscular Mycorrhizal | 6.27 | 5.38 | 7.77 | 3.89 |
|  | Ectomycorrhizal | 0.23 | 0.21 | 0.21 | 0.23 |
|  | Endophyte | 0.07 | 0.03 | 0.06 | 0.04 |
|  | Lichenized | 0.00 | 0.00 | 0.00 | 0.00 |
| Other | Other | 13.99 | 10.96 | 11.98 | 12.98 |
| CK: non-straw retention treatment, SR: sugarcane straw retention treatment. 0-20, 20-40 represent soil samples at 0-20, 20-40 cm depths. | | | | | |


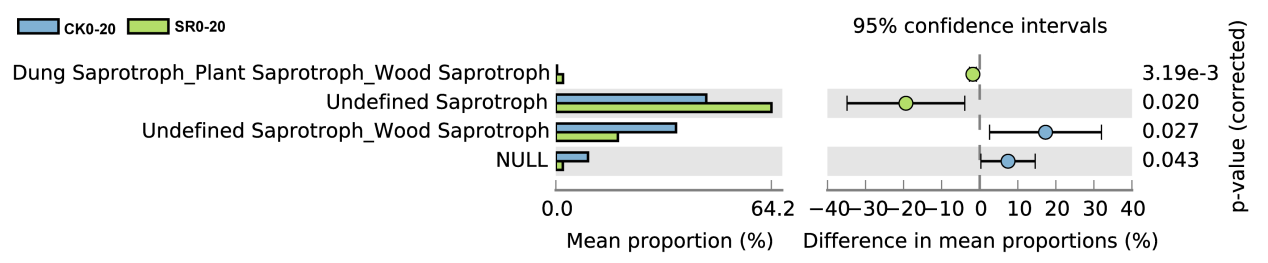


**Figure S1.** Extended error bar graphs indicate the significant difference of fungal functional guilds with control and straw retention treatment in topsoil (0-20 cm) (p < 0.05, average proportion, n = 3). The points depict variations between the "CK0-20" and "SR0-20", the values on the right-hand indicate the p-values obtained from the Welch t-test. CK, control; SR, sugarcane straw retention.


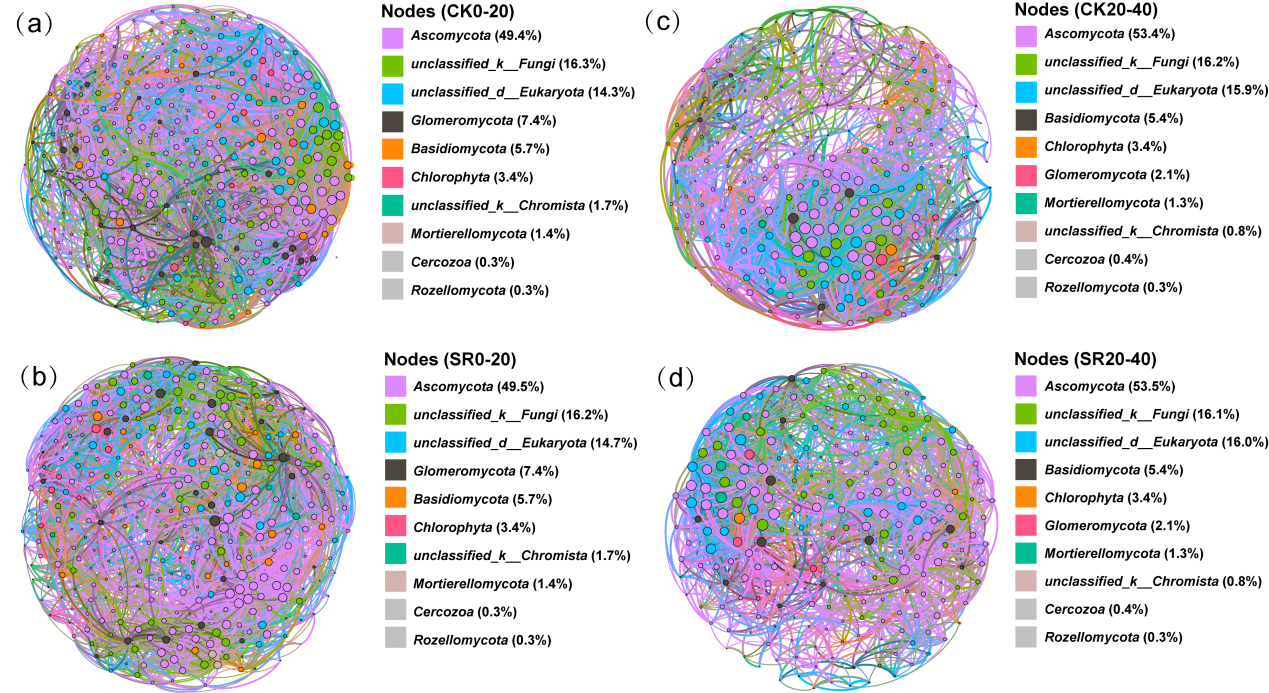


**Figure S2.** Co-occurrence networks with (a) CK0-20, (b) SR0-20, (c) CK20-40, and (d) SR20-40 of the fungal communities in the soil samples. CK, control; SR, sugarcane straw retention.

| **Table S7** Properties of fungal co-occurrence networks. | | | | | | | | |
| --- | --- | --- | --- | --- | --- | --- | --- | --- |
| **Network indexes** | **CK** | **SR** | **0-20 cm** | **20-40 cm** | **CK0-20** | **CK20-40** | **SR0-20** | **SR20-40** |
| Total nodes | 292 | 299 | 350 | 239 | 350 | 239 | 350 | 239 |
| Total edges | 410 | 412 | 641 | 511 | 4979 | 3538 | 5244 | 2798 |
| Positive edges (%) | 82.68 | 77.18 | 85.8 | 90.4 | 62.28 | 65.05 | 54.43 | 65.82 |
| Negative edges (%) | 17.32 | 22.82 | 14.2 | 9.59 | 37.72 | 34.95 | 45.57 | 34.18 |
| Average degree (avgK) | 2.81 | 2.76 | 3.66 | 4.28 | 28.45 | 29.61 | 29.97 | 23.44 |
| Average clustering coefficient (aveCC) | 0.37 | 0.43 | 0.7 | 0.71 | 0.54 | 0.63 | 0.56 | 0.57 |
| Average path distance (GD) | 7.49 | 6.5 | 6.43 | 8.05 | 2.75 | 2.78 | 2.79 | 2.81 |
| Modularity (no. of modules) | 0.97 (130) | 1.04 (150) | 1.61 | 1.03 | 2.14 | 1.57 | 11.76 | 1.75 |
| CK: non-straw retention treatment, SR:sugarcane straw retention treatment. 0-20, 20-40 represent soil samples at 0-20, 20-40 cm depths. | | | | | | | | |

| **Table S8** Variation in fungal functional group compositions of fungal communities at the genus level. | | | | | |
| --- | --- | --- | --- | --- | --- |
| **Treatments** | **Phylum** | **Order** | **Genus** | **Betweeness centrality** | **Trophic Mode** |
| CK | Ascomycota | Pleosporales | *Alternaria* | 4835.73 | Pathotroph-Saprotroph-Symbiotroph |
|  |  | Hypocreales | *unclassified*_*o_*_*Hypocreales* | 4640.57 | not found |
|  |  | Branch06 | *unclassified*_*o*__*Branch06* | 4544.00 | not found |
|  |  | Glomerellales | *Plectosphaerella* | 4447.67 | Pathotroph |
| SR | Ascomycota | Hypocreales | *Trichoderma* | 4964.67 | Saprotroph |
|  |  | Pezizales | *Scutellinia* | 4636.00 | Saprotroph |
|  |  | Glomerellales | *Plectosphaerella* | 4477.38 | Pathotroph |
|  | Glomeromycota | Glomerales | *Claroideoglomus* | 3186.22 | Symbiotroph |
| 0-20 | Basidiomycota | Agaricales | *Clitopilus* | 2511.26 | Saprotroph |
|  |  | Auriculariales | *Auricularia* | 2341.00 | Saprotroph |
|  | Ascomycota | Sordariales | *Staphylotrichum* | 2157.52 | Saprotroph |
|  |  | Pleosporales | *Pyrenochaetopsis* | 1632.32 | Pathotroph-Saprotroph-Symbiotroph |
| 20-40 | Basidiomycota | Polyporales | *Abortiporus* | 2252.50 | Saprotroph |
|  | Chlorophyta | Trebouxiales | *Trebouxia* | 2036.10 | not found |
|  | Ascomycota | Branch06 | *unclassified*_*o_*_*Branch06* | 1726.08 | not found |
|  |  | Pleosporales | *unclassified*_*f*__*Didymellaceae* | 1634.06 | not found |
| CK: non-straw retention treatment, SR:sugarcane straw retention treatment. 0-20, 20-40 represent soil samples at 0-20, 20-40 cm depths. | | | | | |

| **Table S9** Variation in fungal functional groups compositions of fungal communities at the order level. | | | |
| --- | --- | --- | --- |
| **Treatments** | **Phylum** | **Order** | **Trophic Mode** |
| CK | Ascomycota | Pleosporales | Saprotroph; Pathotroph |
|  |  | Hypocreales | Pathotroph-Saprotroph-Symbiotroph |
|  |  | Branch06 | not fund |
|  |  | Glomerellales | Pathotroph |
| SR | Ascomycota | Hypocreales | Pathotroph-Saprotroph-Symbiotroph |
|  |  | Pezizales | Saprotroph; Symbiotroph |
|  |  | Glomerellales | Pathotroph |
|  | Glomeromycota | Glomerales | Symbiotroph |
| 0-20 | Basidiomycota | Agaricales | Saprotroph; Pathotroph-Saprotroph |
|  |  | Auriculariales | Saprotroph |
|  | Ascomycota | Sordariales | Saprotroph; Pathotroph-Saprotroph |
|  |  | Pleosporales | Saprotroph; Pathotroph |
| 20-40 | Basidiomycota | Polyporales | Saprotroph |
|  | Chlorophyta | Trebouxiales | Not fund |
|  | Ascomycota | Branch06 | Not fund |
|  |  | Pleosporales | Saprotroph; Pathotroph |
| CK: non-straw retention treatment, SR:sugarcane straw retention treatment. 0-20, 20-40 represent soil samples at 0-20, 20-40 cm depths. | | | |

**
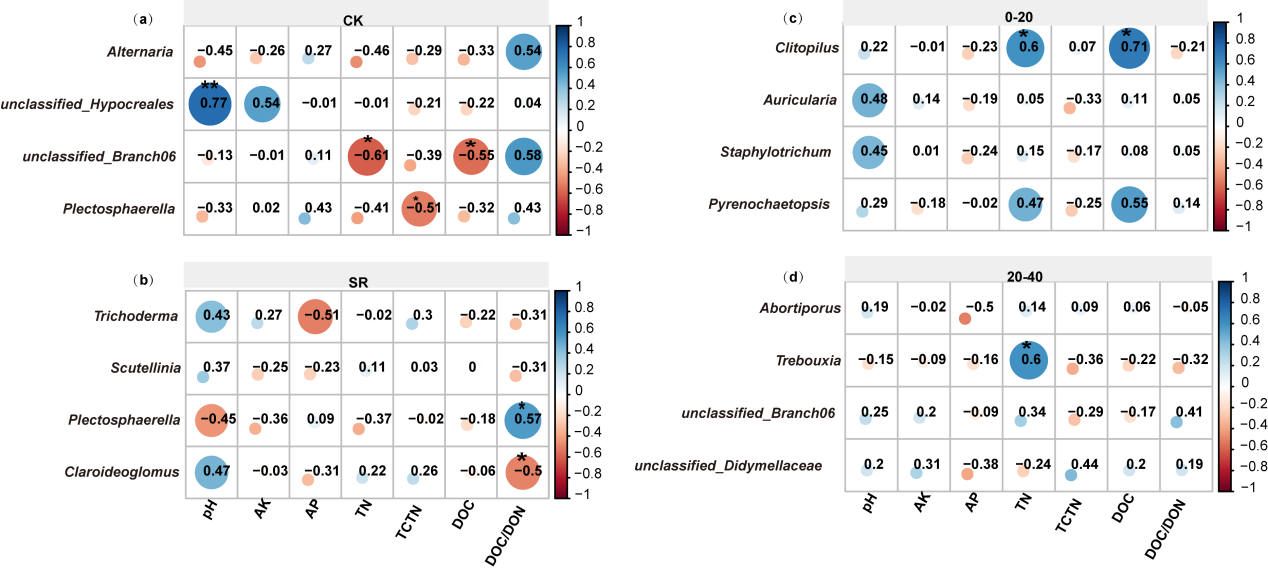

Figure S3.** Pearson’s correlation coefficients of soil physiochemical properties and the keystone taxa of a network (genus), (a) non-straw retention, (b) straw retention, and (c) topsoil (0-20 cm), and (d) subsoil (20-40 cm) depths. The heatmap cells marked by “*” or “**” are statistically significant: * p < 0.05 and ** p < 0.01.
